# Predictive Factors for Clean Intermittent Catheterization after Intravesical OnabotulinumtoxinA Injections in Women with Overactive Bladder: a Danish Retrospective Cohort Study

**DOI:** 10.1007/s00192-024-05960-8

**Published:** 2024-11-07

**Authors:** Meryam El Issaoui, Sophia Elissaoui, Marlene Elmelund, Niels Klarskov

**Affiliations:** 1 Department of Obstetrics and Gynecology, Herlev and Gentofte University Hospital, Borgmester Ib Juuls Vej 1, 16. Etage, 2730 Herlev, Denmark; 2 Department of Clinical Medicine, University of Copenhagen, Copenhagen, Denmark; 3 Department of Gynecology and Obstetrics, Amager and Hvidovre Hospital, Hvidovre, Denmark

**Keywords:** Clean intermittent catheterization, OnabotulinumtoxinA, Overactive bladder syndrome, Post-void residuals, Urgency urinary incontinence, Urinary retention

## Abstract

**Introduction and Hypothesis:**

We aimed to evaluate the clean intermittent catheterization (CIC) rate in women undergoing their first OnabotulinumtoxinA (BTX-A) treatment and to investigate factors predictive of initiating CIC.

**Methods:**

This was a retrospective cohort of women, who had their first BTX-A treatment for symptoms of overactive bladder (OAB) syndrome, with a pretreatment urodynamic study (UDS). We reviewed demographic, medical and gynecological history, UDS, pretreatment bladder diaries, objective examinations, BTX-A treatment details, and post-void residual (PVR) reports in the electronic medical record. Botox® Allergan 100 International Units were injected into the detrusor at 10–20 sites. Statistical analyses included univariate and multivariate logistic regression analyses.

**Results:**

We included 397 women. Median age was 68 (Q1–Q3: 54–76) years. CIC rate was 8.6% (*n* = 34) following the first BTX-A treatment. Urgency urinary incontinence (UUI) reduced the risk of undergoing CIC (OR 0.30, 95% CI 0.09–0.97). A bladder capacity of 500 ml or greater in the bladder diary increased the risk of CIC (OR 2.46, 95% CI 1.06–5.70), whereas reported leakages were associated with a decreased risk of CIC (OR 0.24, 95% CI 0.10–0.57). Multivariate logistic regression analysis showed that anterior colporrhaphy (OR 3.71, 95% CI 1.52–9.06) and 10-ml increments in median maximum cystometric capacity (OR 1.03, 95% CI 1.00–1.06) predicted CIC, whereas UUI was a protective factor for CIC (OR 0.23, 95% CI 0.07–0.79).

**Conclusions:**

A history of anterior colporrhaphy, large bladder capacity, and absence of incontinence episodes in bladder diary or UDS were risk factors for CIC after the first BTX-A treatment.

## Introduction

Intradetrusor OnabotulinumtoxinA (BTX-A) injections are a well-established third-line treatment for overactive bladder (OAB) syndrome. BTX-A treatment is effective for patients with OAB syndrome and urgency urinary incontinence (UUI) who have not been adequately managed by prior anticholinergic and ß3-adrenoceptor agonist therapy. This effect has been demonstrated in large randomized clinical trials (RCTs) [1–4]. Nearly 1,000 BTX-A treatments are performed annually at our tertiary Urogynecology Clinic, and numbers are increasing.

Common adverse effects of BTX-A treatment are urinary tract infections (UTIs) and urinary retention. A recent meta-analysis found a significantly increased risk of urinary retention, with a relative risk (RR) of 8.89 (95% CI: 4.39–17.6), following BTX-A treatment compared with placebo [1]. A previous review of adverse events following BTX-A treatment reported clean intermittent catheterization (CIC) rates between 6.2 and 43.8% [5]. Existing literature is heterogeneous with smaller populations and lacks a standardized definition for initiating CIC. Further elucidation is needed to identify risk factors for urinary retention and CIC after BTX-A treatment [5]. CIC is a procedure associated with discomfort and the risk of UTI [6]. In a clinical setting, it would be important to predict the risk of CIC from the patients’ medical and urogynecological history and examination, including urodynamic studies (UDS). Predicting CIC associated with BTX-A would improve patient selection, help to manage patient expectations, and aid in patient counseling and referral for BTX-A treatment. Some patients opt out of BTX-A treatments because of the risk of urinary retention [7]. Therefore, the ability to provide more specific counseling is of clinical interest.

In this study, we aimed to evaluate the CIC rate in women undergoing their first intradetrusor BTX-A treatment and to investigate factors predictive of initiating CIC.

## Materials and Methods

Our retrospective cohort study was approved by the Capital Region’s Knowledge Center for Data Reviews (ID: R-21013125). Data extraction of this cohort of women, who had their first BTX-A treatment at the Department of Gynecology and Obstetrics between January 2015 and December 2020, was from the electronic medical journal system Epic Systems. Patient data, including demographics, medical and gynecological history, UDS, pretreatment bladder diary, objective examinations, information on BTX-A treatments, and information on post-void residual (PVR) reporting were reviewed in the medical record. All collected data were stored in the online database Research Electronic Data Capture (REDCap).

### Study Population

Women older than 18 years, who had their first BTX-A treatment in our Urogynecology Clinic between January 2015 and December 2020, were identified. Referral for BTX-A treatment was based on complaints of urgency or UUI. In our clinical practice, patients may receive BTX-A treatment if clinically indicated, irrespective of UDS-confirmed detrusor overactivity.

Inclusion criteria were a pretreatment UDS available for assessment in the medical record.

Most patients receiving BTX-A treatment in our clinic have undergone UDS. Patients in this study were included based on the availability of UDS records. Women with a PVR requiring CIC before the first BTX-A treatment were excluded.

### OnabotulinumtoxinA Treatment

Patients were treated with OnabotulinumtoxinA (BTX-A), Botox® Allergan. BTX-A was injected into the detrusor at 10–20 sites under cystoscopic guidance, sparing the trigone. Treatments were administered by urogynecologists or certified nurses, either in an operating theatre or our outpatient clinic, depending on the need for sedation during injections. Perioperative or prophylactic antibiotics were not routinely administered. Antibiotics were prescribed if the clinician identified an increased risk of post-treatment UTI or observed perioperative signs or symptoms of a UTI.

### Bladder Diary and Urodynamic Studies

Patients referred to our clinic usually fill out a 2-day bladder diary. Available pretreatment bladder diary parameters were assessed by calculating the average of the 2-day results. Parameters from the bladder diaries included: fluid intake, number of voids, void volumes, and leakage episodes.

Post-void residual from free flow was assessed by ultrasound during gynecological examination or by bladder scan.

Urodynamic studies were performed by a certified nurse and followed the International Continence Society’s (ICS) recommendations. The Medical Measurement Systems (MMSl Solar System), was used with the following equipment: Dual Lumen Catheters 7F (reference number DLC-7D), Laborie Abdominal Pressure Catheter size 9F (reference number RPC-9), Meritrans DTXPlus transducers (Reference number 682018), MMS filling tube for Nexam (reference number OBS 275/C (33252)), and Laborie Pressure Transmission Tubing-Clear (Reference number PTTA-C).

In our clinic, UDS were initially performed with women seated, followed by a standing-position measurement if indicated by the initial test results. Filling cystometry was conducted using tempered physiological saline 0.9% at a filling rate of 50 ml per minute. During the filling phase, the following parameters were measured: bladder sensation, first desire to void, cystometric detrusor overactivity, volume at first detrusor contraction, detrusor overactivity incontinence, maximum detrusor pressure during filling, maximum cystometric capacity (MCC), and urodynamic stress incontinence. PVR was measured using a sterile single-use catheter. UDS results presented in this study were based on a renewed analysis of UDS by the authors.

### Post-Void Residual Reporting

Patients undergoing BTX-A treatments are routinely informed to contact our outpatient clinic if they experience adverse effects, primarily bladder-emptying problems. Some patients contact our clinic regarding UTI symptoms, whereas others consult their general practitioner for UTI assessment and treatment. Follow-up assessments of PVR are scheduled for selected patients based on the health care provider's evaluation.

For the outcome analysis, patients were included in the CIC group if post-treatment PVR required clean intermittent catheterization (CIC) and in the non-CIC group otherwise.

Our clinical guideline defines significant PVR and indication for post-treatment CIC as PVR greater than 200 ml, with symptoms such as incomplete bladder emptying, suprapubic pain, UTIs, or urgency, or asymptomatic PVR greater than 350 ml. Patients with PVR between 200 and 350 ml are offered expectant treatment with follow-up assessments as needed.

### Statistical Analysis

Data were analyzed in R Studio, version 2022.07.1. Results were reported as medians with interquartile ranges (Q1–Q3) and percentages. Differences in continuous variables between the two outcome groups were assessed using the Wilcoxon rank sum test. Categorical variables were compared using Pearson’s Chi-squared test or Fisher’s exact test. We conducted univariate and multivariate logistic regression analyses to examine the odds of CIC. All statistical assessments were considered significant at a level of *p* < 0.05.

## Results

We included 400 women who had pretreatment UDS. Of these, 3 were excluded at baseline because they did CIC in the pretreatment period. In total, 397 were included in the analyses. Overall median age at first BTX-A treatment was 68 (Q1–Q3: 54–76) years.

There were no significant differences in baseline demographics and characteristics between the CIC group and the non-CIC group (Table 1). Perioperative antibiotics were administered to 46 of the women (11.6%) and were associated with an increased risk of CIC (OR 3.17, 95% CI 1.38–7.31). In the post-treatment period, 41 women (8.5%) had UTIs requiring antibiotic treatment, and among them, 19 (46.3%) underwent CIC. UTI was associated with an increased risk of CIC (OR 19.6, 95% CI 8.80–43.81).

**Table 1:** Baseline characteristics in the clean intermittent catheterization (CIC) group and the non-CIC group

Variable	*N*	Missing (*n*)	CIC group	Non-CIC group	*p* value
Age, median (Q1–Q3)	397	0	66.5 (49.3–73.8)	69 (55–76)	0.353*
BMI (kg/m^2^), median (Q1–Q3)	397	0	27.0 (24.8–30.5)	28.4 (24.6–32.7)	0.169*
Chronic diseases, *n* (%)	334	0	28 (82.4)	306 (84.3)	0.767**
Diabetes, *n* (%)	57	0	3 (8.8)	54 (14.9)	0.448***
Cardiovascular diseases, *n* (%)	204	0	15 (44.1)	189 (52.1)	0.640**
Obesity, *n* (%)	161	0	11 (32.4)	150 (41.3)	0.403**
CNS diseases, *n* (%)	54	0	3 (8.8)	51 (14.0)	0.600***
Other chronic diseases ^a^, *n* (%)	179	0	18 (52.9)	161 (44.4)	0.336**

*CIC* clean intermittent catheterization, *BMI* body mass index, *CNS* central nervous system

*Wilcoxon rank sum test

**Pearson’s Chi-squared test

***Fisher’s exact test

^a^Hematological diseases, other endocrine disorders, metal disorders, lung diseases, cancer, autoimmune diseases, gastrointestinal diseases, kidney diseases, rheumatic disorders

### Clean Intermittent Catheterization Rate

Out of 397, a total of 34 (8.6%) underwent CIC treatment. Median time interval between the first BTX-A treatment and PVR diagnosis was 13.5 (Q1–Q3: 6.8–21.3) days. Two women were diagnosed with PVR when they returned for their second BTX-A treatment, 3 months and 2 years after their initial BTX-A treatment. In the CIC group, 16 (47.1%) had a PVR volume exceeding 300 ml, 10 (29.4%) had a PVR between 200 and 299 ml, and 7 (20.6%) had a PVR lower than 200 ml at diagnosis. One woman in the CIC group had no PVR volume documented in the medical record. During the intervention period, one woman required a temporary indwelling catheter. In the CIC group, another woman was hospitalized with pyelonephritis 1 month after treatment. CIC duration was 8 weeks or more for 16 women (47.1%), 5 (14.7%) performed CIC for 4–8 weeks, and 7 (20.6%) for less than 4 weeks. CIC duration was not documented in the medical records for 6 women (17.6%). Of the women who underwent CIC, 15 (44%) had more than one BTX-A treatment, whereas 19 (55.9%) did not continue BTX-A treatment.

### Gynecological History and Pretreatment Bladder Diary

Women with a history of UUI had a 70% reduced risk of undergoing CIC (OR 0.30, 95% CI 0.09–0.97). In the CIC group, 9 (26.5%) had a history of anterior colporrhaphy, which was associated with an increased likelihood of CIC (OR 3.86, 95% CI 1.65–8.99). Similarly, 9 (26.5%) women had previously undergone a midurethral sling (MUS) procedure, which was predictive of CIC in the univariate analysis (OR 2.75, 95% CI 1.20–6.29). A history of a hysterectomy procedure was associated with CIC (OR 2.13, 95% CI 1.01–4.50; Table 2).

**Table 2:** Risk of clean intermittent catheterization (CIC) medical history

					Univariate analysis*	
Variable	*N*	Missing (*n*)	CIC group	Non-CIC group	OR 95% CI	*p* value
Age/10-year change	397	0			0.88 (0.97–1.01)	0.264
Hysterectomy, *n* (%)	86	0	12 (35.3)	74 (20.4)	2.13 (1.01–4.50)	0.048
Partus, *n* (%)	392	5	31 (91.2)	318 (87.6)	1.30 (0.38–4.45)	0.676
0	43		3 (8.8)	40 (11.0)	1.00 (ref.)	
1	47		5 (14.7)	42 (11.8)	1.59 (0.36–7.08)	0.545
≥ 2	286		24 (70.6)	262 (72.2)	1.22 (0.35–4.24)	0.753
Cesarean section, *n* (%)	27	10	4 (11.8)	23 (6.3)	1.91 (0.62–5.90)	0.259
Spasmolytics, *n* (%)	371	0	33 (97.1)	338 (93.1)	2.34 (0.31–17.87)	0.411
UUI, *n* (%)	379	0	30 (88.2)	349 (96.1)	0.30 (0.09–0.97)	0.045
SUI, *n* (%)	208	14	14 (41.2)	194 (53.4)	0.59 (0.29–1.22)	0.155
MUI, *n* (%)	198	14	12 (35.3)	186 (51.2)	0.52 (0.25–1.08)	0.080
Cystocele, *n* (%)	348	49				
POP-Q stage						
0–1	296		24 (70.6)	272 (74.9)	1.00 (ref.)	
≥ 2	52		5 (14.7)	47 (13.1)	1.21 (0.44–3.32)	0.717
Uterine prolapse, *n* (%)	324	73				
POP-Q stage						
0–1	316		27 (79.4)	289 (79.6)	1.00 (ref.)	
≥ 2	8		1 (2.9)	7 (1.9)	1.53 (0.18–12.89)	0.696
Rectocele,* n* (%)	339	58				
POP–Q stage						
0–1	300		26 (76.5)	274 (75.5)	1.00 (ref.)	
≥ 2	39		3 (8.8)	36 (9.9)	0.88 (0.25–3.05)	0.838
Previous urogynecological surgery (*N* = 108, missing = 6)						
MUS, *n* (%)	51		9 (26.5)	42 (11.6)	2.75 (1.20–6.29)	0.016
Bulking, *n* (%)	21		1 (2.9)	20 (5.5)	0.52 (0.07–4.00)	0.529
Anterior colporrhaphy, *n* (%)	40		9 (26.5)	31 (8.5)	3.86 (1.65–8.99)	0.002
Posterior colporrhaphy, *n* (%)	5		0	5 (1.4)	0 (0.00–Inf)	0.989

*CIC* clean intermittent catheterization, *UUI* urgency urinary incontinence, *SUI* stress urinary incontinence, *MUI* mixed urinary incontinence, *MUS* midurethral sling, *POP-Q* Pelvic Organ Prolapse Quantification, *OR* odds ratio, *CI* confidence interval

*Logistic regression analysis

Pretreatment bladder diaries were available for 367 women. A bladder capacity of 500 ml or greater increased the risk of CIC more than two times (OR 2.46, 95% CI 1.06–5.70). Reported leakages in the bladder diary were associated with a 76% lower risk of CIC (OR 0.24, 95% CI 0.10–0.57; Table 3).

**Table 3:** Free post-void volume and bladder diary characteristics in the clean intermittent catheterization (CIC) and non-CIC groups

					Univariate analysis*	
Variable	*N*	Missing (*n*)	CIC group	Non-CIC group	OR (95% CI)	*p* value
Free PVR/10-ml increments, median (Q1–Q3)	397	0	28.5 (10–55.3)	30 (10–50)	1.02 (0.9–1.13)	0.766
Bladder diary						
Bladder capacity/10-ml increments, median (Q1–Q3)	367	30	305 (250–500)	300 (220–400)	1.01 (0.98–1.03)	0.562
Bladder capacity categorized/ml, *n* (%)						
0–499			20 (58.8)	286 (78.8)	1.00 (ref.)	
500–1,030			9 (26.5)	52 (14.3)	2.46 (1.06–5.70)	0.035
Micturition, median (Q1–Q3)	367	30	8.5 (7–11)	10 (8–12)	1.02 (0.94–1.11)	0.674
Fluid intake/ml, median (Q1–Q3)	367	30	1,300.0 (1,050.0–1,697.3)	1,500 (1,175–1,825)	1.00 (1.00–1.00)	0.138
Fluid intake categorized/ml, *n* (%)						
0–1,499 ml			21 (61.8)	171 (47.1)	1.00 (ref.)	
≥ 1,500 ml			9 (26.5)	166 (45.7)	0.44 (0.20–0.99)	0.048
Total voided volume/ml, median (Q1–Q3)	367	30	1,451.5 (1,103.8–1,653.8)	1,488.0 (1,130.0–1,876.5)	1.00 (1.00–1.00)	0.348
Micturition volume/ml, median (Q1–Q3)	367	30	148 (119.5–218.8)	150 (113.8–192.3)	1.00 (1.00–1.00)	0.437
Incontinence episodes, *n* (%)	325	33	22 (64.7%)	303 (83.5%)	0.24 (0.10–0.57)	0.001
Incontinence episodes, median (Q1–Q3)	324	34	3.5 (2–4)	5.0 (3–8)	0.82 (0.70–0.97)	0.020

*CIC* clean intermittent catheterization, *OR* odds ratio, *CI* confidence interval, *PVR* post-void residual

*Logistic regression analysis

### Urodynamic Studies

In our cohort, 318 women (80.1%) had urodynamic detrusor overactivity, which showed no difference between the CIC group and the non-CIC group (*p* = 0.579). The CIC group had significantly greater MCC (350.0 ml [Q1–Q3: 253.0–441.5], compared with the non-CIC group (290 ml (Q1–Q3: 174.0–400.0), *p* = 0.017). An MCC greater than 350 ml significantly increased the risk of CIC compared with an MCC lower than 200 ml (OR 3.65, 95% CI 1.20–11.12). Cystometric incontinence (comprising both detrusor overactivity incontinence and urodynamic stress incontinence) showed an insignificant trend toward reducing the CIC risk by 50% (OR 0.50, 95% CI 0.25–1.03, *p* = 0.059; Table 4).

**Table 4:** Urodynamic parameters in the clean intermittent catheterization (CIC) and non-CIC groups

					Univariate analysis*	
Urodynamic parameter	*N*	Missing	CIC group	Non-CIC group	OR (95% CI)	*p* value
First desire/ml, median (Q1–Q3)			147 (111–237)	138 (94–212)	1.00 (1.00–1.01)	0.453
MCC/10-ml increments, median (Q1–Q3)	397	0	350.0 (253.0–441.5)	290 (174–400)	1.03 (1.00–1.06)	0.016
MCC categorized/ml, *n* (%)	397	0				
200			4 (11.8)	103 (28.4)	1.00 (ref.)	
200–349			12 (35.3)	133 (36.6)	2.32 (0.73–7.41)	0.154
≥ 350			18 (52.9)	127 (35.0)	3.65 (1.20–11.12)	0.023
Cystometric DO, *n* (%)	318	0	26 (76.5)	292 (80.4)	0.79 (0.34–1.82)	0.579
Vol. first detrusor contraction/ml, median (Q1–Q3)	318	0	225.0 (91.3–335)	160 (100–280)	1.00 (1.00–1.01)	0.158
Cystometric incontinence, *n* (%)	279	0	19 (55.9)	260 (71.6)	0.50 (0.25–1.03)	0.059
Mixed cystometric incontinence, *n* (%)	63	0	5 (14.7)	58 (16.0)	0.91 (0.34–2.44)	0.846
Cystometric detrusor incontinence, *n* (%)	242	0	18 (52.9)	224 (61.7)	0.70 (0.34–1.41)	0.318
Cystometric stress incontinence, *n* (%)	99	0	6 (17.6)	93 (25.6)	0.62 (0.25–1.55)	0.308
Cystometric PVR/10-ml increments, median (Q1–Q3)	397	0	65.0 (0–170.0)	33.0 (0–134.5)	1.00 (1.00–1.00)	0.531
Cystometric PVR categorized/ml, *n* (%)	397	0				
0–49			15 (44.1)	201 (55.4)	1.00 (ref.)	
≥ 50			19 (52.9)	162 (44.6)	1.57 (0.77–3.19)	0.211
Cystometric PVR categorized/ml, *n* (%)	397	0				
0–99			20 (58.8)	249 (68.6)	1.00 (ref.)	
≥ 100			14 (41.2)	114 (31.4)	1.53 (0.75–3.13)	0.247

*MCC* maximum cystometric capacity, *DO* detrusor overactivity, *PVR* post-void residual, *OR* odds ratio, *CI* confidence interval

*Logistic regression analysis

In a multivariate logistic regression analysis, previous anterior colporrhaphy (OR 3.71, 95% CI 1.52–9.06) and 10-ml increments in MCC (OR 1.03, 95% CI 1.00–1.06) were identified as predictive factors for CIC, whereas UUI was a protective factor for CIC (OR 0.23, 95% CI 0.07–0.79). The association between MUS and CIC lost statistical significance, although a trend indicated an increased risk of CIC in the multivariate analysis (Table 5).

**Table 5:** Risk indicators associated with clean intermittent catheterization (CIC) after intradetrusor onabotulinumtoxinA (BTX-A) treatment

	Univariate analysis*		Multivariate analysis*	
Variable	OR (95% CI)	*p* value	OR (95% CI)	*p* value
UUI	0.30 (0.09–0.97)	0.045	0.23 (0.07–0.79)	0.019
MUS	2.75 (1.20–6.29)	0.016	1.96 (0.81–4.71)	0.134
Anterior colporrhaphy	3.86 (1.65–8.99)	0.002	3.71 (1.52–9.06)	0.004
MCC/10-ml increments	1.03 (1.00–1.01)	0.016	1.03 (1.00–1.06)	0.038

*UUI* urgency urinary incontinence, *MUS* midurethral sling, *MCC* maximum cystometric capacity, *OR* odds ratio, *CI* confidence interval

*Logistic regression analysis

## Discussion

### Clean Intermittent Catheterization Rate

In our retrospective cohort of 397 women, we report a CIC rate of 8.6% following the first BTX-A treatment. CIC rates following the first BTX-A treatment have been reported to be as high as 48.9% [1, 5, 8–11]. A recent meta-analysis comprising 10 RCTs evaluating BTX-A compared with placebo reported an RR of 8.89 (95% CI 4.39–17.6) for developing urinary retention or CIC [1]. Another meta-analysis reported an RR of 13.99 (95% CI 5.71–34.30) for urinary retention in patients receiving BTX-A treatment with 100 Units [12]. RCTs have reported lower CIC rates. Nitti et al. reported a CIC rate of 6.1% in an RCT with a PVR indication similar to that of our study [4], whereas Chapple et al. reported a CIC rate of 6.9% [2]. Conversely, cohort studies have reported higher CIC numbers. Squires et al. found a 10% CIC rate among 187 women receiving their first BTX-A treatment for idiopathic UUI in a retrospective cohort. Authors defined urinary retention as incomplete bladder emptying, worsened urgency or frequency, difficulty voiding, suprapubic pain, with PVR greater than 150 ml, or PVR greater than 300 ml without bladder symptoms [13]. Osborn et al. reported a CIC rate of 21% in 101 patients (76% women) with idiopathic OAB who had their first BTX-A treatment. CIC indication was based on the patients’ symptoms, with only 24% of those undergoing CIC having a post-treatment PVR [14].

Variability in reported CIC rates in the literature may be due to different definitions of PVR, as concluded in a recent review by Abrar et al. [5].

Among women who initiated CIC in our study, 47.1% presented with a PVR exceeding 300 ml at the time of diagnosis, which is higher than reported in the literature. The RCT by Chapple et al. found that 10.2% of patients requiring CIC had a postoperative PVR volume of 200 ml or greater [2]. Our clinic does not routinely schedule postoperative follow-ups for all women unless the health care provider assesses a potential risk. Furthermore, our criteria for initiating CIC were based on larger post-treatment PVRs, as our clinical guideline defines PVR requiring CIC as PVR greater than 200 ml with clinical symptoms or PVR greater than 350 ml without symptoms. Our criteria for initiating CIC are more conservative than those of many previous studies, which could explain a lower CIC rate in our cohort. Redundant controls could result in unnecessary CIC prescriptions, potentially causing adverse effects that outweigh the risk of missing an elevated PVR. One woman who required CIC in our cohort was admitted with pyelonephritis.

American Urological Association defines non-neurogenic chronic urinary retention as an elevated PVR exceeding 300 ml persisting for at least 6 months and documented on two or more separate occasions [15]. International Urogynecological Association/ICS Joint Report defines chronic urinary retention as a nonpainful bladder with a chronically high PVR greater than 200 ml [16]. The lack of a standardized definition for urinary retention contributes to the lack of consensus on when to initiate CIC.

Women who received perioperative antibiotics, including both intravenous and peroral administration, had a threefold increased risk of undergoing CIC. UTI could potentially confound this association. Abrar et al. reported that patients requiring CIC (43.8%, *n* = 32) had 5.3 times higher odds of developing UTI than those who did not require CIC [6], suggesting that CIC might be associated with UTI due to instrumentation.

Urinary retention could also be a confounding factor for both UTIs and CIC. Perioperative antibiotics administration may be influenced by the health care provider assessing an increased UTI risk, potentially biasing our results through indication bias.

Clean intermittent catheterization was initiated at a median of 13.50 (Q1–Q3 6.8–21.3) days. However, two women were diagnosed with asymptomatic PVR requiring initiation of CIC when they returned for their second BTX-A treatment, 3 months and 2 years after their first treatment respectively. Liberman et al. found that 80% of their patients initiated CIC within 4 weeks [17]. A previous RCT observed a peak in mean PVR around week 2 following BTX-A treatment, followed by a gradual decrease between weeks 4 and 12 [8], whereas CIC was initiated in almost all within 12 weeks in the RCT by Chapple et al. [2].

In our cohort, nearly half of women in the CIC group (47.1%) performed CIC for over 8 weeks, whereas 7 (20.6%) performed CIC in 4 weeks or less. Osborn et al. reported a mean length of urinary retention for 16 weeks in their cohort [14], whereas mean duration of urinary retention has been reported ranging from 9 weeks [3, 15] to 9 months [14, 18–20]. The exact duration of CIC for each woman in our cohort was not determined owing to the retrospective nature of the study and incomplete information in the medical records.

### Gynecological History

Age was not a predictive factor for CIC in our cohort. This contrasts with previous studies that did identify higher age as a predictor of CIC [21]. A high median age in our cohort could explain the lack of association.

We found a significant association between a previous anterior colporrhaphy and an increased likelihood of CIC, which was confirmed in the multivariate analysis. Urinary retention is a well-known and early postoperative adverse effect following anterior colporrhaphy [22, 23]. However, to our knowledge, urinary retention has not been identified in the literature as a long-term adverse effect or as a risk factor for CIC after BTX-A treatment. Our results contradict those of previous studies on adverse events following prolapse surgery, which showed improvements in bladder emptying and reduced PVR volumes [24, 25]. Confounding factors could influence this novel observed association.

Moreover, we found an association between previous MUS and the risk of CIC in the univariate analysis. This association lost statistical significance in the multivariate analysis, although a trend suggesting an increased risk of CIC persisted. No significant association between CIC and a history of urethral bulking procedure was found. Squires et al. reported that women with a history of anti-incontinence procedures for stress urinary incontinence were more likely to experience urinary retention (53% vs 18%, *p* = 0.002), although they did not have higher baseline PVR (32 ml vs 26 ml, *p* = 0.3) in a cohort of 187 women undergoing BTX-A treatment [13]. However, the anti-incontinence procedure types were not specified in the study. Voiding dysfunction is common following MUS but is often transient and self-limiting [26]. Prolonged catheterization for more than 2–4 days postoperatively owing to urinary retention after MUS has been reported to be 5.6% in a retrospective cohort of 17,030 patients [27]. In our cohort, 10.1% of women had previously undergone an anterior colporrhaphy, and 26.5% of the CIC group had undergone this procedure. Similarly, 12.8% underwent MUS, and 26.5% of the CIC group had undergone this procedure. These findings provide novel and valuable results and should be considered when advising women with a history of previous urogynecological surgery.

Women who had undergone hysterectomy before their first BTX-A treatment had an increased risk of CIC. This association was also reported by Abrar et al. (OR 4.55, 95% CI 1.09–18.87) in their cohort of 74 women with a CIC rate of 43.8% [6].

### Pretreatment Post-Void Residual

Post-void residual was not a significant predictor for CIC, whether adjusted for MCC or analyzed as a continuous or categorized variable. An increased pretreatment PVR is a well-known risk factor for post-treatment PVR requiring CIC [2, 5, 14, 28]. Kuo et al. found a baseline PVR of 100 ml or greater to predict CIC (OR 9.9, 95% CI 7.2–44.7) [28]. Our results contrast with those of previous studies that identified pretreatment PVR as a predictor of CIC. The absence of a significant association between pretreatment PVR and post-treatment CIC in our study could be explained by low pretreatment PVR measurements. All of the women included had a pretreatment PVR lower than 100 ml in the UDS, emphasizing that we carry out a careful assessment before referring our patients for BTX-A treatment. Squires et al. reported similar results to those of our study, with no association between pretreatment PVR and post-treatment urinary retention (25 ± 27 ml vs 46 ± 52 ml, *p* = 0.12) [13].

### Urodynamic Studies

Urodynamic studies are considered the gold standard for investigating lower urinary tract dysfunction [29].

In our study, the CIC group had a significantly greater MCC than the non-CIC group (350.0 ml [Q1–Q3 253.0–441.5] vs 290.0 ml [Q1–Q3 174.0–400.0], *p* = 0.017). A 10-ml increment in MCC increased the risk of CIC by 3% (OR 1.03, 95% CI 1.00–1.06), and MCC of 350 ml or greater increased the risk of CIC by more than threefold (OR 3.65, 95% CI 1.20–11.12, *p* = 0.023). Our results are supported by a previous study that reported similar associations [14]. However, Squires et al. found no differences in MCC between the groups (359 ± 150 vs 321 ± 105, *p* = 0.64) [13].

### Bladder Diary

Elevated bladder capacity and reported leakages in pretreatment bladder diaries were predictive of CIC, aligning with the risk factors identified through UDS. Based on these findings, the results of pretreatment UDS provided limited additional information on the risk of CIC compared with the bladder diary. Our findings suggest that bladder diaries could serve as valuable assessment tools when counseling women undergoing BTX-A treatments.

### Strengths and Limitations

The strengths of our study lie in the assessment of a consecutive single-center cohort of women who had BTX-A treatment at our department. We evaluated an unselected cohort with baseline UDS as the most important inclusion criterion, thereby presenting clinically relevant results. Existing studies have reviewed cohorts with UDS as a subgroup. Our cohort only comprised women, whereas numerous other studies included a mixed population of women and men, possibly contributing to higher CIC rates. Men undergoing BTX-A treatment are at a higher risk of post-treatment CIC [10]. In addition, this study is to our knowledge the first to identify an association between CIC and previous anterior colporrhaphy, likely because it is the first to be conducted on a large female population.

Limitations include the retrospective nature of the study. CIC reporting was based on journal notes from the medical records, with missing documentation and information. Also, we may have underestimated the CIC rate because we do not systematically assess for PVR, potentially missing patients with asymptomatic PVR greater than 350 ml.

## Conclusion

In this retrospective cohort comprising 397 women undergoing their first BTX-A treatment, we report a CIC rate of 8.6%. Women with a history of anterior colporrhaphy were at a risk of CIC nearly four times higher. We confirm an association between increased MCC and the risk of CIC. Women with UUI were at a lower risk of initiating CIC. Elevated bladder capacity and a lack of reported leakages in pretreatment bladder diaries predicted CIC, aligning with the risk factors identified through UDS.

## Data Availability

Data is unfortunately not available due to the Danish Data Protection Agency’s rules because the data contains sensitive information.
